# Photomorphogenic and Biochemical Effects of Radiation and Nitrate Availability on the Red Alga *Plocamium cartilagineum*

**DOI:** 10.3390/plants14071121

**Published:** 2025-04-03

**Authors:** Bruna Rodrigues Moreira, Julia Vega, Marta García-Sánchez, Cristina González-Fernández, Antonio Avilés, José Bonomi-Barufi, Félix L. Figueroa

**Affiliations:** 1 Laboratório de Ficologia, Departamento de Botânica, Universidade Federal de Santa Catarina, Florianópolis 88040-900, Brazil; jose.bonomi@gmail.com; 2 Centro Experimental Grice Hutchisnon, Instituto Andaluz de Biotecnología y Desarrollo Azul (IBYDA), Universidad de Málaga, Lomas de San Julián, 2, 29004 Málaga, Spain; juliavega@uma.es (J.V.); martagarciasanchez81@gmail.com (M.G.-S.); cristinagonfdez@gmail.com (C.G.-F.); aviles@uma.es (A.A.)

**Keywords:** *Plocamium cartilagineum*, photosynthesis, photoreceptors

## Abstract

Non-photosynthetic photoreceptors detecting different wavelength ranges in the UV and visible region of spectra may trigger algal acclimation and homeostasis. We studied *Plocamium cartilagineum* responses based on the saturation of photosynthesis by Amber light and supplementation by different light qualities, applying an experimental design able to simulate a daily cycle in a fully automated system. Thalli were exposed to Amber, Amber + UV-A, Amber + Blue and Amber + Green radiation treatments under two nitrate levels (60 and 240 μM) for enrichment lasting two weeks. *P. cartilagineum* photosynthesis and biochemistry were measured during different experimental periods. Photosynthesis showed only slight variations, emphasizing that other response variations could be activated by photomorphogenic pathways. Nitrate assimilation was higher in the treatments containing blue and green lights, potentially caused by increasing nitrate reductase activity. Photosynthetic pigments and mycosporine-like amino acids were affected over the two weeks, being mostly influenced by UV-A and blue radiations with the highest nitrate concentration. The shinorine content of thalli under blue radiation with 240 μM of nitrate increased at day 7, possibly modulated by a blue light photoreceptor. The increase in the bioactive compounds in the short-term by specific light qualities under optimal photosynthetic performance was found to be a relevant biotechnological strategy.

## 1. Introduction

Photosynthetic organisms are affected significantly by sunlight. The light-harvesting complex captures the energy of photosynthetic active radiation (PAR), transferring it to photosystem II, activating an electronic flow at the electron transport chain. This electron flow is driven by photosynthetic photochemical reactions and can regulate carbon fixation, nitrate assimilation, antioxidant responses, among others in algal metabolism, due to the use of reduced ferredoxin, NADPH and photosynthetic ATP in the assimilation processes [1]. However, besides photosynthesis, light can also trigger non-photosynthetic photoreceptors (or light-sensitive proteins), thus activating other physiological responses. Photoreceptors are capable of continuously monitoring light and triggering signal transduction chains generating cellular and molecular responses [2]. Light quantity and quality signaling can be provided for these photoreceptors, controlling photomorphogenesis, phototropism and photoperiodism. The processes and compounds associated with light photoreception vary among different macroalgal groups [3,4].

Distinct groups of photoreceptors have been described in the different groups of algae, including phytochromes, cryptochromes, phototropins, photoactive yellow protein, blue and UV-light receptors [5]. Three types of rhodopsin-like receptors associated with other proteins and carotenoid vesicles were found in *Chlamydomonas* sp., and they were organized into a light-perception system. The beating pattern of this green alga flagella can be modulated by rhodopsin photoreceptors, which are light-gated ion channels. It drives the algae in the direction of light, improving photosynthesis and survival [6,7]. Eyespots formed by photoreceptors and carotenoids are also found in *Euglena* spp. [8]. Monoclonal antibodies were used to detect phytochrome-like proteins in macroalgae [9,10], later being confirmed in other publications on diverse micro and macroalgal species [4,11]. Photoreceptors may detect changes in the light pattern of a daily cycle, altering pigment content and leading to micro and macroalgae photoacclimation. The synthesis of chlorophyll and phycobiliproteins was linked to phytochrome or phytochrome-like photoreceptor by the studies of López-Figueroa and Niell, 1989 [12] and López-Figueroa et al., 1990 [13]. These authors provided pulses of red and far-red radiation in *Corallina elongata* and *Porphyra umbilicalis*, two red macroalgae species. Furthermore, photoprotective compounds were also controlled by triggering blue and UV photoreceptors in macroalgae [14,15], highlighting mycosporine-like amino acids (MAAs). These molecules absorb UV radiation, avoiding photodamage and are considered a photoprotection mechanism. Around 30 types of MAAs have been identified in algae [16].

In addition to radiation, nutrient availability plays an important role in photomorphogenic and photosynthetic algal responses. *Ulva rigida* cultivated under nitrate supply presented increasing ETR_max_ values, and it also decreased algal photoinhibition after exposure to high solar radiation [17]. The combination of radiation and nutrients may also result in interesting modulations. For example, a photoprotective effect of NO_3_^−^ was found on *Gracilaria tenuistipitata*, where the synthesis of MAAs reduced the negative effects of UV radiation [18]. Also, a positive effect of N-supply in the photosynthesis of *Gracilaria conferta* under UV exposure was shown by Figueroa et al., 2010 [19] due to the increase in phycoerythrin and phycocyanin synthesis. Blue light also caused the increase in nitrate absorption by stimulating nitrate reductase and glutamine synthetase activities, stimulating the production of amino acids and pigments [20].

Investigations of photomorphogenesis in plants or algae have included diverse experimental designs. Since the first studies determining phytochrome activity in seed germination of lettuce, other research has been performed to understand how photoreceptors affect plants and algae development [21]. Some authors simply exposed the algae to continuous and fixed amounts of light, where the photoperiod is set to switch on or off [22,23,24]. Algal and plant physiology and biochemistry can be modulated by varying photoperiods [25]. Other studies complemented PAR with low intensity levels for stimulating photoreceptors besides photosynthesis. In this sense, photosynthesis was saturated with the amber color provided by yellow low pressure sodium lamps (SOX light), while other algal responses were induced by the addition of low amounts of other monochromatic lights [15,26,27]. However, none of these studies focused on variations in light intensity during light photoperiod exposure, simulating a diel light increase/decrease in laboratory conditions.

*Plocamium cartilagineum* (Plocamiaceae) is a red macroalga, found in intertidal and subtidal zones in tropical and temperate areas around the world, awaking a great biotechnological interest due to its high chemical complexity. A huge variety of compounds like organic acids, proteins, polysaccharides, terpenes, lipids, halogenated compounds, pigments and mycosporine-like amino acids are produced by this species [28,29,30]. Those molecules extracted from *P. cartilagineum* are reported by many authors as photoprotective, antioxidant, insecticide, virucidal, anticancer molecules, among others [29,31,32,33]. All those mentioned bioactivities highlight *P. cartilagineum* as a potential alga to be cultivated and used by pharmaceutical, cosmetic and nutraceutical industries. Moreover, the production of specific compounds may be enhanced by the utilization of novelty cultivation strategies, such as, for example, those applying distinct radiation quality and quantity to photoreceptor stimulations.

**Figure 1:**
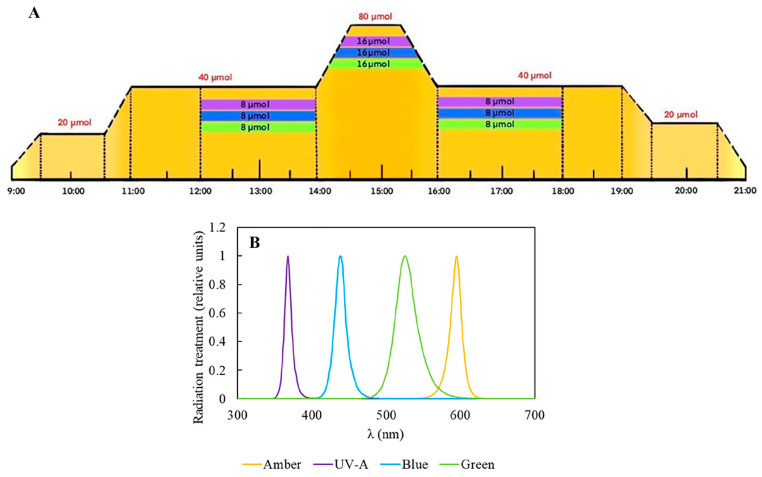
Experimental design performed in the Aralab Bioclimatic chamber. (**A**) Representative scheme of the experimental design containing the different radiation qualities and intensities. Amber was employed during the whole daily cycle and is represented by yellow color; meanwhile, complementary radiations are restricted to certain periods of the day and are represented by violet, (UV-A), Blue and Green. (**B**) Relative absorption spectrum of the lamps employed in the experiment.

In this study, we applied different radiation treatments associated with two nitrogen-source concentrations to influence *Plocamium cartilagineum*’s physiology and biochemistry for two weeks. To achieve this, algae were cultivated in climate-controlled chambers, which allowed the simulation of a daily cycle pattern and its light fluctuation (Figure 1).

## 2. Results

### 2.1. Photosynthetic Parameters

The photosynthetic parameters of *P. cartilagineum* exposed to various treatments are presented in Table 1 and Figure S1. The experiment aimed to promote the photoreceptor’s modulation by exposing algae thalli to low irradiance from different radiation qualities (UV-A, Blue or Green) under similar photosynthetic rates provided by Amber. Data derived from rapid light curves (Table 1), performed on days 1, 7 and 14 of the experiment, evidenced stable photosynthetic parameters, presenting only slight variability in some of them. On day 7, none of the photosynthetic parameters were affected by the treatments evaluated. Although Anova (Table S1) indicated that F_v_/F_m_ was affected by the interaction between radiation and nitrate concentration, the Newman–Keuls post hoc test did not detect any variation among the treatments. So, we found an average of 0.531 ± 0.06 for F_v_/F_m_, 0.063 ± 0.02 for α_ETR_, 6.1 ± 1.0 μmol e^−^ m^−2^ s^−1^ for ETR_max_ and 115.5 ± 73.1 μmol photons m^−2^ s^−1^ for E_k_. α_ETR_ and F_v_/F_m_ did not present significant differences among the treatments after 14 days, with an average of 0.076 ± 0.01 and 0.511 ± 0.06, respectively. Nutrient concentration only affected E_k_ values observed on day 14, which presented higher values of E_k_ on treatments receiving 240 μM of nitrate than on treatments containing 60 μM. ETR_max_ was significantly influenced by radiation and nutrient availability, presenting the highest values on thalli exposed to Amber + Blue (240 μM of nitrate) and Amber (60 μM of nitrate), corresponding, respectively, to 8.0 ± 1.5 and 7.7 ± 0.9 µmol e^−^ m^−2^ s^−1^, and the lowest value on thalli under Amber + Green (60 μM of nitrate) treatment, corresponding to 4.6 ± 0.7 µmol e^−^ m^−2^ s^−1^.

A similar in situ ETR variation was observed during the experimental period in *P. cartilagineum* thalli (Figure S1). Radiation treatments did not influence in situ ETR responses on every experimental day (Table S2). Slight reductions in ETR were observed at specific times in the day: on day 1 at 18:20, on day 5 at 09:45 and at 18:20 and on day 12 at 09:45, considering treatments containing 240 μM of nitrate when compared to those containing 60 μM of nitrate.

### 2.2. Nutrient Uptake

**Figure 2:**
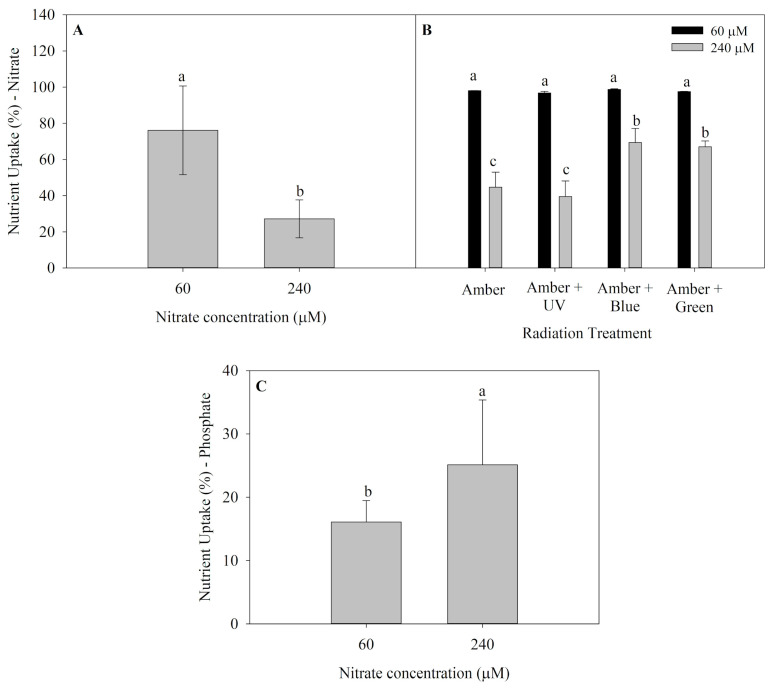
Nutrient uptake in *Plocamium cartilagineum* thalli cultivated under the influence of different treatments. (**A**) Nitrate uptake on day 7 from algae cultivated using different nitrate concentrations. (**B**) Nitrate uptake on day 14 from *P. cartilagineum* thalli cultivated under different radiation treatments and nitrate concentrations. (**C**) Phosphate uptake from *P. cartilagineum* thalli on day 14 using different nitrate concentrations. Different letters indicate significant differences among the treatments on days 7 and 14, independently.

Nutrient uptake (NU) of *P. cartilagineum* for both nitrate and phosphate are presented in Figure 2. Algae thalli cultivated in saltwater containing 60 μM of nitrate presented the highest nutrient uptake in the first week of the experiment (Figure 2A), incorporating 76.1 ± 24.5% of nitrate available, which is equivalent to around 45.6 μM of nitrate. An interaction between radiation treatments and nitrate availability was observed in the second week (Figure 2B), and the nitrate uptake was also higher in algae grown with 60 μM of nitrate. Furthermore, it was noticed that algae grown under treatments containing Amber + Blue and Amber + Green radiations and receiving 240 μM of nitrate exhibited a higher nitrate uptake when compared to thalli grown using only Amber and Amber + UV-A radiation. Phosphate uptake (average of 39.5 ± 8.1%) was not affected by any factors during the first week. However, algae grown under 240 μM of nitrate absorbed more phosphate than those cultivated with 60 μM of nitrate during the second week (Figure 2C).

### 2.3. Photosynthetic Pigments

**Figure 3:**
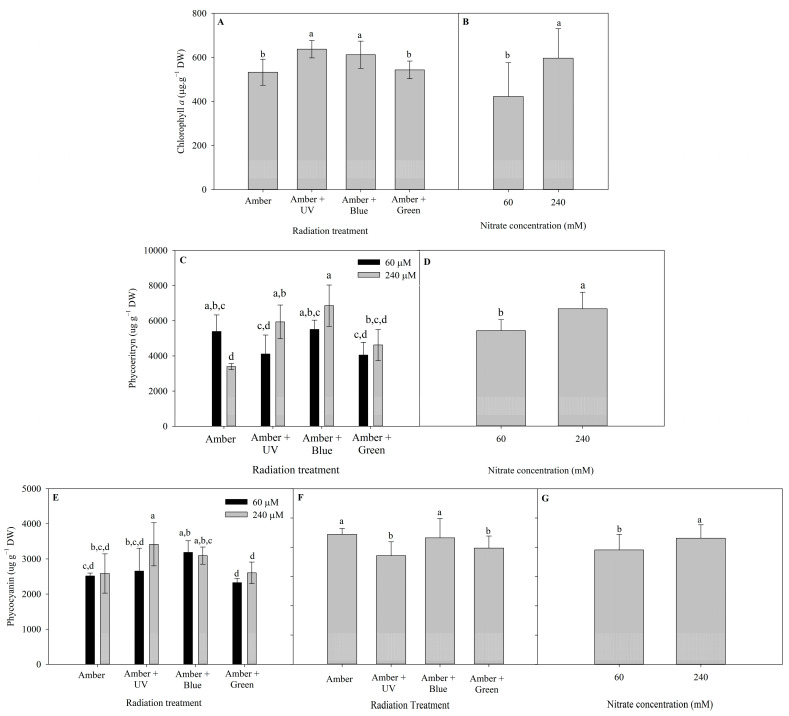
Photosynthetic pigments determined in *Plocamium cartilagineum* thalli on the different experimental treatments. (**A**) Chlorophyll *a* content on algae thalli on day 7 influenced by radiation treatments. (**B**) Chlorophyll *a* content on algae thalli on day 14 influenced by nitrate concentration. (**C**) Phycoerythrin content on algae thalli on day 7 influenced by the interaction between radiation treatment and nitrate concentration. (**D**) Phycoerythrin content on algae thalli on day 14 influenced by nitrate concentration. (**E**) Phycocyanin content on algae thalli on day 7 influenced by the interaction between radiation treatment and nitrate concentration. (**F**) Phycocyanin content on algae thalli on day 14 influenced by radiation treatment. (**G**) Phycocyanin content on algae thalli on day 14 influenced by nitrate concentration, independently. Different letters indicate significant differences (*p* < 0.05) among the treatments at distinct times.

The different patterns of pigment contents of *P. cartilagineum* (Chlorophyll *a* (Chl *a*), Phycoerythrin (PE) and Phycocyanin (PC) contents) after 7 and 14 days of the experiment are presented in Figure 3. Radiation treatments influenced Chl *a* content during the first week and nitrate concentration influenced Chl *a* during the second week (Table S3). The highest contents of Chl *a* on day 7 were detected in algae thalli under Amber + UV-A (637.3 ± 39.6 µg. g DW^−1^) and Amber + Blue treatments (612.1 ± 61.9 µg. g^−1^ DW). On day 14, algae grown under 240 μM of nitrate exhibited a higher content of Chl *a* (597 ± 134.4 µg. g^−1^ DW) than algae grown under 60 μM of nitrate (423 ± 153.3 µg. g^−1^ DW).

There is not a clear pattern in the production of PE on algal thalli from day 7 (Figure 3C). The interaction of radiation treatment and nitrate concentration evidenced a higher level of PE in Amber (60 μM of nitrate) when compared to Amber (240 μM of nitrate). On the other hand, the highest nitrate concentration increased PE production when using Amber + UV-A radiation and Amber + Blue. On day 14, the pigment production was enhanced using 240 μM of nitrate (6674 ± 938 µg. g^−1^ DW). In the case of PC content, on day 7, only the use of 240 μM of nitrate combined with Amber + UV-A radiation increased PC content, compared with the use of 60 μM of nitrate. On day 14, the effects of radiation treatments (Figure 3F) and nitrate concentration (Figure 3G) were observed independently. The utilization of Amber and Amber + Blue enhanced PC production, as well the use of 240 μM of nitrate.

### 2.4. Phenolic Compounds

**Figure 4:**
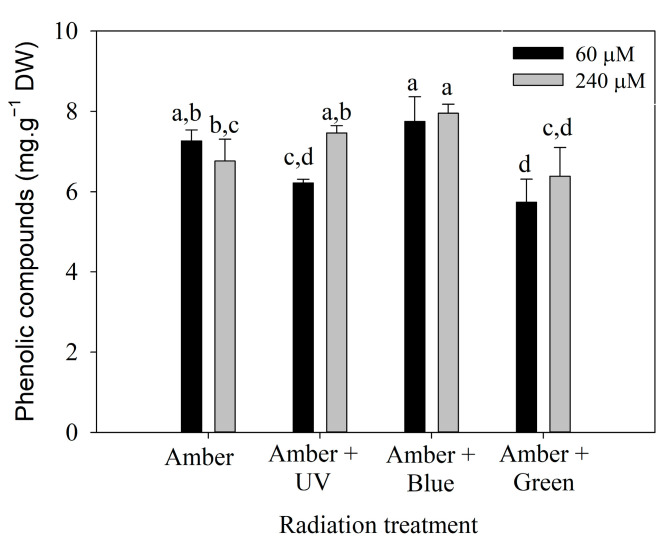
Phenolic compounds measured in *Plocamium cartilagineum* thalli on day 7 of the experiment resulted from the interaction between radiation treatment and nitrate concentration. Different letters indicate significant differences (*p* < 0.05) among the treatments.

Polyphenol content on day 7 (Figure 4) was influenced by the interaction between radiation treatment and the nitrate concentration (Table S3). It can be observed that *P. cartilagineum* exhibited a higher polyphenol content when exposed to Amber + Blue radiation compared to the other three treatments. Phenolic compounds were effectively stimulated by the combination of Amber + Blue radiation in both nitrate concentrations. Although ANOVA analysis of day 14 (Table S4) indicates a significant difference in polyphenol content influenced by radiation treatment, the post hoc analysis did not confirm the pattern.

### 2.5. Mycosporine-like Amino Acids

**Figure 5:**
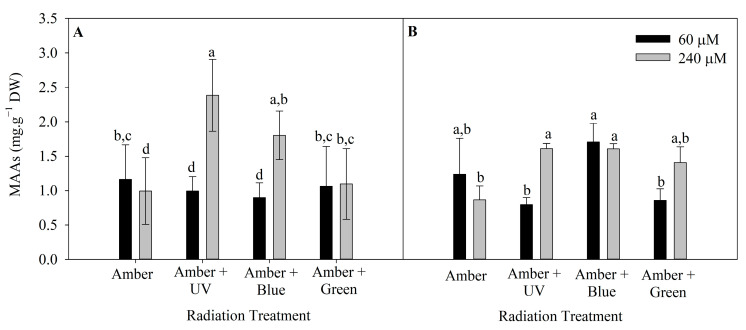
Total mycosporine-like amino acids on *Plocamium cartilagineum* thalli exposed to different treatments. (**A**) MAAs content on thalli at day 7, influenced by radiation treatment. (**B**) MAAs content on day 14, influenced by the interaction between radiation treatment and nitrate concentration. Different letters indicate significant differences (*p* < 0.05) among the treatments at distinct times.

The level of MAAs in *P. cartilagineum* was influenced by radiation treatment and nutrient concentration in both weeks of the experiment (Tables S3 and S4). The highest concentration of MAAs was 2.4 ± 0.5 mg. g^−1^ DW, in algal thalli cultivated under Amber + UV radiation and 240 μM of nitrate (Figure 5). This cultivation condition was more efficient in stimulating MAAs production/accumulation than all the other treatments, except for algae cultivated under Amber + Blue, which also contained 240 μM of nitrate.

**Figure 6:**
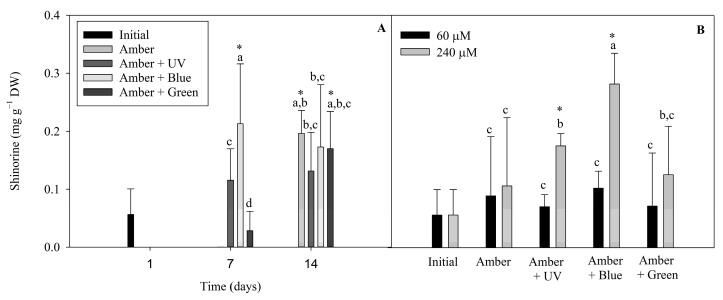
Shinorine levels detected on *P. cartilagineum* thalli exposed to the different treatments, as follows: (**A**) shinorine levels resulted from the interaction between radiation and time, and (**B**) shinorine levels resulted from the interaction between radiation and nitrate concentration. Different letters indicate significant differences among the treatments. * Indicates significant difference from the initial samples using Student’s *t*-test.

The following different MAAs found in *P. cartilagineum* were identified and quantified by using HPLC chromatograms associated with their specific standards: palythine, traces of asterine-330 and shinorine. We also found two unidentified peaks, with similar retention times, absorbing UV radiation at 332 nm. Shinorine levels varied during the two weeks of the experiment (Figure 6). Shinorine concentration was directly influenced by the interaction between radiation and time, and by radiation and nitrate concentration, independently (Table S5). Regarding the first interaction (Figure 6A), the highest contents of shinorine were detected on the treatments using Amber + Blue on day 7, Amber on day 14 and Amber + Green on day 14. This showed higher values than the initial concentrations measured on day 1, indicating that shinorine levels were increased by these treatments. Figure 6B evidenced that shinorine in *P cartilagineum* was increased in treatments using Amber + Blue associated with a higher nitrate availability. An increase in shinorine was also observed on thalli exposed to Amber + UV-A radiation containing 240 µM of nitrate when compared to those containing 60 µM of nitrate. Both treatments, Amber + Blue and Amber + UV-A, both containing 240 µM of nitrate, had their content of shinorine increased 4.7 and 2.8 times, respectively, when compared to thalli from the first day of the experiment (0.06 ± 0.04 mg g^−1^ DW).

## 3. Discussion

In this paper, we focused on evaluating the biochemical parameters of *P. cartilagineum* using, as an experimental design, a fully automated system able to simulate an entire daily cycle, to assess the influence of radiation treatments and nutrient concentration on *Plocamium cartilagineum* thalli for 2 weeks. Our study employed the use of Aralab bioclimatic chambers. This methodology helps to elucidate how algae develop acclimation mechanisms to survive different environmental changes throughout the day, including possible photomorphogenic responses. Furthermore, the automated systems facilitate the execution of longer experiments in controlled conditions when compared to lights on or off systems. The diel mechanism allows the algae to receive gradual irradiances during the day. Then, acclimation procedures associated with gradual brightness increase or decrease can be properly considered here.

Daily cycle variations have an impact on algal physiology and biochemistry, and most of the studies focusing on this parameter comprise field studies under solar radiation [30,34,35,36]. Although some of these studies are performed for a longer period, the measurements require a complex infrastructure and are subject to adverse conditions, such as the presence of clouds and heatwaves. Another important fact is that as the sun emits a broad spectrum of radiation, it would be much more complicated to fully evaluate the activity of specific photoreceptors, requiring immunological or molecular approaches to ensure that these compounds are being activated. Hence, this justifies our choice to use Amber as photosynthetic radiation, as it is similar to the strategy used by other authors [27,37,38,39]. In addition, Amber radiation proved to be efficient in stimulating photosynthesis in plants by inducing a higher quantum yield and increasing dry mass more than the red radiation [40]. Considering the complementary radiation UV-A, Blue and Green, it is estimated that low fluences varying from 1 to 20 μmol photons m^−2^ s^−1^ are enough to activate photoreceptors [3,41,42].

As mentioned in the methodology section, the experimental design was primarily planned to stimulate photoreceptor activity by saturating photosynthesis rates without causing photoinhibition. In this sense, it was observed that although some variability occurred within the photosynthetic parameters, we registered only minor differences among them. This was the expected result as we purposefully used Amber as the photosynthetic radiation to maintain the stable photosynthetic rate. Complementary radiation was also added to stimulate the activity of photomorphogenic photoreceptors [15] and genes mediated by a cAMP in the transduction chain [43,44]. The absence of significant differences in F_v_/F_m_ values indicates that *P. cartilagineum* did not undergo photoinhibition due to the treatment’s exposure.

Regarding pigment content, UV and Blue radiation were the most efficient in stimulating chlorophyll production in the first week of the experiment, while in the second week, only a higher nitrate concentration affected the pigment concentration positively. López-Figueroa and Niell, 1990 [13], studying *Ulva rigida*, *Corallina elongata*, *Plocamium cartilagineum* and *Porphyra umbilicalis*, evidenced that continuous blue light exposition for the period of 6 h stimulated chlorophyll *a* synthesis. On the other hand, green and red lights were more efficient in stimulating phycoerythrin and phycocyanin, respectively, in the studied red algae. Ben Ghedifa et al., 2021 [39] reported that green light stimulated both R-phycoerythrin and soluble proteins in the red algae *Gracilaria gracilis*. Thus, green light stimulated phycoerythrin accumulation, which is a pigment with maximal absorption in green light (565 nm). However, in our study, PE accumulation of *P. cartilagineum* was not stimulated by green light. It is relevant to mention that PE has another additional peak at 498 nm, close to the blue region of the spectra. We hypothesize that chlorophyll *a* and PE content regulation in *P. cartilagineum* is mainly determined by a Blue receptor and indicates an ecological adaptation, considering that *P. cartilagineum* usually occurs in subtidal and deeper water conditions and, in clear waters, Blue prevails with increasing depth. A high nitrogen availability may also result in the increase in phycobiliproteins and total proteins of the species, as found in our study. Previous studies have already shown the nitrogen regulation of biliprotein contents, such as Figueroa et al., 2010 [19], Navarro et al., 2014 [45] and Yu et al., 2022 [46]. The increase in pigments by blue light is related to the stimulation of nitrogen metabolism, both in nitrate uptake and nitrate reduction by nitrate reductase activity [20,22,47].

Many field studies and laboratory experiments, using constant photoperiods, try to comprehend how the production of MAAs is affected by radiation and nutrient availability [15,18,24,36]. The production of MAAs using systems able to simulate a full daily cycle in laboratories is still uncommon. The Aralab bioclimatic chamber utilized in our experiment allows for the proper management of light quality and quantity along the photoperiod, and this can be a good strategy for modulating responses regulated for a short amount of time (hours, for example). Diel light regulation is an advantageous strategy, as it presents different responses according to the variation in irradiances.

The experiment indicated that the treatment with Amber + Blue and Amber + UV-A radiations, associated with a high nitrate availability, enhanced the production of MAAs in *P. cartilagineum*. Korbee et al., 2005 [24], studying *Porphyra leucosticta* under blue and white treatments, provided with a high source of nitrogen, also observed an increase in MAAs production. Studies indicate that blue radiation promotes nitrate incorporation as well as stimulation of nitrate reductase and glutamine synthetase activities [20]. Nitrate assimilation was incremented both by blue and by green complementary irradiances when *P. cartilagineum* received more nutritional supply. This pattern was not evident when a lower amount of N was available. Then, it is possible to infer that nitrate assimilation in *P. cartilagineum* is light-controlled only when a higher quantity of inorganic nitrogen is available. Nutrient assimilation can be regulated by nutrient availability coupled with radiation quality, mainly through blue light photoreceptors (cryptochromes). Additionally, UV and Blue radiation have been reported to stimulate the activity of photoreceptors by increasing total MAAs levels and the proportion of specific MAAs [15,24,48].

An interesting achievement was the increase in shinorine levels, mainly on the 7th day under UV-A and Blue treatments. Krabs et al., 2004 [49] suggest that the activity of one or two UV-A photoreceptors in the red algae *Chondrus crispus* modulates the production of shinorine in this species. They also noticed the same effect using blue light, a fact that may be correlated to the photoregulation of pterin synthesis, a chromophore presented in the UV-A/Blue photoreceptors.

The decrease in total MAA content after 14 days has already been reported by other authors [50,51], possibly motivated by photodegradation caused by UV long term exposure. However, when analyzing the variation in MAA composition, it can be observed that certain MAAs decreased whereas other MAAs increased. In this study, shinorine is maintained at a high level after 14 days under high nitrogen levels, therefore, nitrate seems to be crucial for maintaining high levels of MAAs. Perhaps, during the interconversion of MAAs in the pathway, certain MAAs are favored under high nitrogen levels compared to others. Interestingly, during an ozone hole episode in sub-Antarctic area in Chile, i.e., increased UV-B radiation, Navarro et al., 2014 [45] observed that UV-A absorbing MAAs as shinorine and palythine maintained similar levels to those before ozone depletion, whereas mycosporine-glycine, an UV-B absorbing MAA, had increased. Thus, the proportion of different MAAs can change in the short term (hours and days) and is influenced by both UV radiation and nitrate availability [45,52].

In conclusion, Blue and UV-A radiations, coupled with a higher availability of nitrogen, were found to be relevant for regulating the bioactive compounds of *P. cartilagineum*, mainly after 7 days. Thus, from a biotechnological point of view, the harvest for extracting bioactive compounds is more favorable at 7 d than at 14 d. This is advantageous as it can provide an increase in bioactive compounds in the short term. MAAs and phycobiliproteins were significantly increased with complementary radiation, indicating that photoreceptors-like cryptochromes can act in the photoregulation of species metabolism. This is mainly relevant when considering biotechnological approaches for eliciting valuable compounds for industrial purposes, especially for cosmeceutical applications such as more environmentally and health friendly sunscreen [16,53], although the cultivation of *Plocamium* spp. is still a challenge that must be overcome.

## 4. Materials and Methods

### 4.1. Biological Material and Acclimation

The red macroalga *Plocamium cartilagineum* (Linnaeus) P.S.Dixon (Plocamiales, Rhodophyta) was collected at La Herradura Beach, Granada, Spain (36°44′17″ N, 3°45′01″ W) in January 2022. Algae were quickly transported to the Instituto de Biotecnología y Desarrollo Azul (IBYDA) in Malaga city (36°43′12″ N, 4°25′13″ W), where the experiment was conducted. Algae thalli were manually cleaned for debris and epiphytes. Acclimation was performed by exposing *P. cartilagineum* to 20 μmol m^−2^ s^−1^ of amber radiation (λ = 590 nm), using an Amber lamp provided by LEDs, in artificial seawater produced by the addition of sodium chloride in distilled water (salinity 36), and supplemented with 50 μM of potassium nitrate and 3 μM of sodium phosphate dibasic, which was then maintained at 18 °C for a week. All acclimation processes and the experiment were conducted in an FITOCLIMA 25000 PLH Climatic Chamber (Aralab, Lisbon, Portugal).

### 4.2. Experimental Design

For the experiment, 3 g L^−1^ of fresh *P. cartilagineum* were placed into polymethylmethacrylate cylinders, which were transparent to UV-radiation. To evaluate the potential effect of light quality and nutrient availability on photosynthetic performance and biochemical properties, the *P. cartilagineum* was exposed to 4 different types of radiation provided by LEDs: Amber (λmax 590 nm), Amber + UV-A (λmax 365 nm), Amber + Blue (λmax 420 nm) and Amber + Green (λmax 520 nm); two concentrations of potassium nitrate (60 μM and 240 μM) and 3 μM of sodium phosphate dibasic, for 2 weeks. These treatments configured an initial N:P ratio of 20:1 and 80:1, respectively. The water in the cylinders was changed on the 1st and 7th day of the experiment, followed by nutrient addition. Water samples were obtained at different interval periods in order to evaluate the algae’s nutrient uptake (see details below). In this sense, the experiment consisted of the exposure of *P. cartilagineum* to 8 different treatments with 3 replicates in each one, as exhibited in Figure 1. The experiment was designed to simulate the pattern of a sunny day without clouds. This involved an increase in radiation until midday, in a photoperiod of 12:12 h, hereafter considered from 9:00 to 21:00 as the light period. Amber was used as a saturating photosynthetic light in all treatments. In this way, when the Amber light was switched on, at 9:00, it increased gradually until it reached 20 μmol m^−2^ s^−1^ at 9:30, and remained at this irradiance for one hour (9:30–10:30). Next, another gradual increment of 20 μmol m^−2^ s^−1^ was provided during 30 min, achieving 40 μmol m^−2^ s^−1^ at 11:00 and remaining at this intensity for the 3 next hours. Then, a new gradual increment of Amber light was supplied, reaching up to 80 μmol m^−2^ s^−1^ at 14:30 h and remaining at this level for one hour. Then, from 15:30 to 16:00, the irradiance gradually decreased until it reached 40 μmol m^−2^ s^−1^. Again, the samples continued to receive this amount of light for the subsequent 3 h and, from 19:00 to 19:30, a new gradual drop was applied, until the value of 20 μmol m^−2^ s^−1^ was reached. Samples received this irradiance for one hour, and at 20:30 the system started to dim the light automatically, decreasing irradiance from 20 to 0 within 30 min. Then, from 21:00 to 9:00 the next day, the samples remained in darkness. The total daily Amber dose was 288.7 kJ m^−2^. The irradiance values chosen for the experiment were determined by previously incubating algal thalli in various increasing Amber irradiances for 5 min. The electron transport rate (ETR) was determined as described below, and light curves were fitted to a model by Eilers and Peters, 1988 [54], ensuring the determination of saturating irradiance (see details of this procedure below). In that case, the value found for irradiance saturation was close to 95 μmol m^−2^ s^−1^, but we opted to utilize a slightly lower value as the maximal irradiance along the daily cycle to avoid the risk of driving the algae into photoinhibition. Besides being exposed to Amber, three groups were exposed to complementary radiation (UV-A, Blue, Green) from 12:00 to 18:00. In this sense, complementary radiation of 8 μmol m^−2^ s^−1^ was initially provided from 12:00 to 14:00, and then it was gradually doubled for 30 min until it reached 16 μmol m^−2^ s^−1^, and it remained at this level for one hour (14:30 to 15:30). Complementary irradiance also decreased by half to 8 μmol m^−2^ s^−1^, then remaining at this amount for two hours more until it was switched off at 18:00 (Figure 1). The total daily doses were 335.7 kJ m^−2^ for Amber + UV-A, 340.9 kJ m^−2^ for Amber + Blue and 335.5 kJ m^−2^ for Amber + Green treatments.

### 4.3. Photosynthetic Performance

Photosynthetic parameters were measured during the experiment by in vivo chlorophyll *a* fluorescence using a Mini-PAM (Walz, Effeltrich, Germany). The effective quantum yield (Y(II)) and maximum quantum yield (F_v_/F_m_) of photosystem II were assessed as described by Figueroa et al., 2014 [55] on days 1, 5, 8 and 12, in four different periods throughout the day: 8:20 (F_v_/F_m_), 9:45, 15:00 and 18:20 h.

Y(II) data were obtained three times during the day and they were used to calculate in situ electron transport rates. Rapid light curves (RLC) were performed on the 1st, 7th and 14th days during the period from 9:00 to 14:00 h, using the Mini-PAM fluorometer connected to the WinControl-3 software (Walz, Effeltrich, Germany). During this interval, radiation treatments remained constant to avoid interfering with the test due to the variation in irradiances. Algal samples from each treatment were acclimated during 15 min in darkness and subsequently exposed to the following increasing irradiances: 0, 25, 45, 66, 90, 125, 190, 285, 420, 625, 845, 1150 and 1500 μmol photons m^−2^. s^−1^ of red actinic light. After the incubation of each irradiance, the samples received a saturation pulse. Electron transport rates (ETR) were calculated according to the following equation:

ETR = Y(II) × E × A × 0.15,(1)

where Y(II) corresponds to the effective quantum yield of PSII, E corresponds to the irradiance to which the algae was exposed, and 0.15 is equivalent to the factor for adjusting the irradiance captured and used by photosystem II (PSII) by red macroalgae. A refers to the absorptance calculated by the following equation according to Grzymski et al., 1997 [56] and Figueroa et al., 2003 [57]:

(2) $$A=1-\frac{E_{F}}{E_{T}},$$

where EF corresponds to the irradiance transmitted throughout the algae thalli and ET is the total irradiance, measured with the Li-189 radiometer (LICOR Ltd., Lincoln, NE, USA).

### 4.4. Nutrient Assimilation

The nutrient uptake (NU) was calculated for nitrate and phosphate by considering all conditions evaluated in this experiment. To evaluate nutrient assimilation by algae, water samples were taken as soon as the nutrients were added at day 1 (after adding the nutrients), at day 7 and at day 14 (before water changing and nutrient addition), filtered through 0.45 μM and frozen at −80 °C for further analysis. The remaining content of each nutrient in the water was quantified as described by Grasshoff et al., 1999 [58], using a segmented flow analyzer (SFA; Seal Analytical autoanalyzer QuAAtro). NU was calculated as described by Massocato et al., 2022 [59] using the following formula:

(3) $$\mathrm{NU}\;(\%)=100-\left(\frac{C_{sample}\times 100}{C_{initial}}\right)$$

where *C_sample_* represents the nutrient concentration on the water sample for each treatment and *C_initial_* the initial nutrient concentration added to each treatment.

### 4.5. Photosynthetic Pigments

In order to determine photosynthetic pigments, we used distinct extracts. For Chl *a* quantification, 15 mg of dry algae were macerated with 2 mL of acetone 90% previously prepared with 10% of a saturated solution of C_4_Mg_4_O_12_. The mixture was extracted overnight, at 4 °C in darkness. At the end of the process, the mixture was centrifuged for 10 min, at 4 °C and 10,000 rpm. Chl *a* content on acetonic extracts was calculated according to the methodology of Ritchie, 2008 [60], as described in the equation:

Chl *a* (μg mL^−1^) = 11.4711 × (A_664_ − A_750_) − 1.6841 × (A_691_ − A_750_). (4)

For phycoerythrin (PE) and phycocyanin (PC), the extracts were prepared by macerating 15 mg of dry algae with 2 mL of sodium phosphate buffer pH 6.8. The mixture was incubated overnight at 4 °C in darkness, and subsequently centrifuged during 10 min, at 4 °C and 10,000 rpm. R-PE and R-PC on aqueous extracts were quantified according to the methodology described by Wiley and Neefus, 2007 [61], described in the following equations:

R-PE (mg mL^−1^) = 0.1247 × (A_564_ − A_730_) − 0.4583 × (A_618_ − A_730_), (5)

R-PC (mg mL^−1^) = 0.154 × (A_618_ − A_730_). (6)

### 4.6. Phenolic Compounds

The quantification of phenolic compounds was performed using the same aqueous extract employed for the quantification of phycobiliproteins, as described in item 2.5. After extraction, the content of phenolic compounds was measured using the methodology of Folin–Ciocalteu, 1927 [62]. The reaction was carried out by mixing 100 µL of algal extract with 700 μL of distilled water, 50 μL of Folin–Ciocalteu reagent, and lastly, 150 μL of 20% sodium carbonate (Na_2_CO_3_), which starts the reaction. The mixture was incubated for 2 h at 4 °C. After incubation, the absorbance was measured at 760 nm. The results are expressed in the mg equivalent of phloroglucinol (Ph) per gram of algal dry weight (DW), and were calculated based on a phloroglucinol (6-hydroxy-2, 5, 7, 8-tetramethylchroman-2-carboxylic acid) calibration curve, using the following linear fitting (r^2^ = 0.99):

y = 0.0641x + 0.0174. (7)

### 4.7. Micosporine-like Amino Acids

Mycosporine-like amino acids (MAAs) were extracted by grinding 15 mg of algae dry biomass with 1 mL of 20% methanol. The mixture was incubated for 2 h at 45 °C. After incubation time, the mixture was centrifuged for 10 min, at 10,000 rpm. The supernatant was collected and dried in a vacuum concentrator (Speed-Vac SPD210, Thermo Scientific, Waltham, MA, USA). The dry extract was resuspended in 1 mL of methanol HPLC grade and filtered through a 0.2 µm membrane filter. MAAs analyses were performed using ultra high-performance liquid chromatography uHPLC (1260 Agilent InfinityLab Series, Santa Clara, CA, USA) according to the methodology described by Korbee-Peinado et al., 2004 [48]. The mobile phase used for MAAs separation was composed by aqueous methanol 2.5% (*v*/*v*) and acetic acid 0.1% (*v*/*v*) in water. A total of 30 µL of each sample was injected in a Luna C8 chromatographic column (Phenomenex, Aschaffenburg, Germany). The flow rate was set at 0.5 mL. min^−1^ for a run time of 20 min per sample. MAAs peaks were detected in a photodiode detector at 320 and 330 nm. Isolated MAAs obtained via HPCCC [63] were used as standard, and quantification was conducted based on the molar extinction coefficients (Ɛ) of the MAAs [64]. The results are expressed in mg g^−1^ DW.

### 4.8. Statistical Analysis

Factorial ANOVAs (analysis of variance) were applied for the data obtained on day 7 and on day 14, independently. The independent factors used in ANOVA were radiation treatment and nitrate concentration. The Student–Newman–Keuls post hoc test was used to identify the correlation among the responses, considering a significance level of *p* < 0.05. All statistical analyses were conducted using Statistica 7.0 (StatSoft. Inc., Tulsa, OK, USA) and graphs were created using SigmaPlot 14.0 software (Systat Inc., Palo Alto, CA, USA).

## Figures and Tables

**Table 1:** Photosynthetic parameters of *Plocamium cartilagineum* exposed to different radiation treatments (Amber, Amber + UV-A, Amber + Blue and Amber + Green) and nitrate concentrations (60 μM and 240 μM) on days 7 and 14 of the experiment, obtained from rapid light curves. Data are mean ± SD. Different letters indicate significant differences among α_ETR_, ETR_max_, E_k_ and F_v_/F_m,_ independently.

Treatments			F_v_/F_m_	α_ETR_	ETR_max_	E_k_
Time	Radiation	NO_3_				
Initial	-	-	0.474 ± 0.07	0.054 ± 0.01	4.2 ± 0.7	79.3 ± 5.3
7 days	Amber	60	0.506 ± 0.05	0.054. ± 0.03	5.4 ± 1.4	64.3 ± 8.6
	Amber	240	0.536 ± 0.07	0.07 ± 0.01	6.2 ± 0.2	83.3 ± 8.1
	Amber + UV-A	60	0.526 ± 0.05	0.053 ± 0.02	6.4 ± 0.9	108.3 ± 29.1
	Amber + UV-A	240	0.498 ± 0.07	0.067 ± 0.02	5.8 ± 0.6	88.7 ± 15.2
	Amber + Blue	60	0.492 ± 0.04	0.058 ± 0.03	6.3 ± 1.1	82.5 ± 21.6
	Amber + Blue	240	0.589 ± 0.04	0.072 ± 0.01	6.9 ± 1.1	96.7 ± 23.5
	Amber + Green	60	0.603 ± 0.01	0.076 ± 0.01	6.4 ± 0.9	85.3 ± 17.9
	Amber + Green	240	0.500 ± 0.02	0.048 ± 0.01	6.4 ± 0.4	162.5 ± 12.5
14 days	Amber	60	0.527 ± 0.03	0.082 ± 0.01	7.7 ± 0.9 ^a^	94.1 ± 3.4
	Amber	240	0.466 ± 0.06	0.068 ± 0.01	6.0 ± 0.4 ^a,b^	88.6 ± 12.2
	Amber + UV-A	60	0.492 ± 0.03	0.081 ± 0.01	7.3 ± 1.4 ^a,b^	89.7 ± 19.2
	Amber + UV-A	240	0.500 ± 0.05	0.070 ± 0.01	7.2 ± 0.7 ^a,b^	102.7 ± 13.6
	Amber + Blue	60	0.480 ± 0.06	0.088 ± 0.02	6.5 ± 1.7 ^a,b^	77.6 ± 30.0
	Amber + Blue	240	0.536 ± 0.06	0.074 ± 0.01	8.0 ± 1.5 ^a^	109.8 ± 29.1
	Amber + Green	60	0.551 ± 0.02	0.070 ± 0.01	4.6 ± 0.7 ^b^	65.7 ± 7.5
	Amber + Green	240	0.534 ± 0.10	0.073 ± 0.02	7.5 ± 0.7 ^a,b^	105.5 ± 18.4

## Data Availability

The original contributions presented in this study are included in the article/Supplementary Material. Further inquiries can be directed to the corresponding authors.

## Supplementary Materials

The following supporting information can be downloaded at: https://www.mdpi.com/article/10.3390/plants14071121/s1.

## Abbreviations

The following abbreviations are used in this manuscript:

ANOVA	Analysis of variance
Chl *a*	Chlorophyll *a*
DW	Dry weight
ETR	Electron transport rate
MAA	Mycosporine-like amino acid
NU	Nutrient uptake
PC	Phycocyanin
PE	Phycoerythrin
UV-A	Ultraviolet A radiation
