# Supplementary material for: Photomorphogenic and Biochemical Effects of Radiation and Nitrate Availability on the Red Alga Plocamium cartilagineum
Source: Plants (Basel). 2025 Apr 3;14(7):1121. doi: 10.3390/plants14071121 (PMC11991008; doi:10.3390/plants14071121)
Supplement: Supplementary file 1 [file plants-14-01121-s001.zip › plants-3506180-supplementary.pdf]

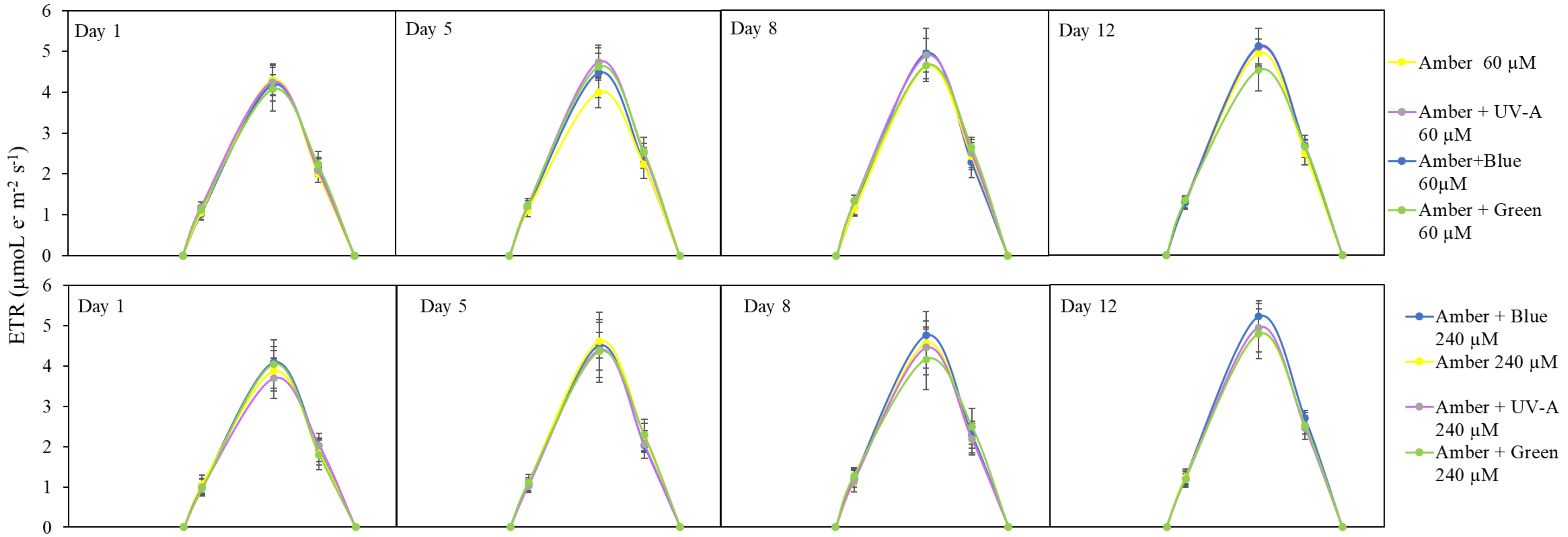

Figure S1. *In situ* Electron transport rate (ETR) measured in *P. cartilagineum* thalli throughout the day, at 08:20, 09:45, 15:00 and 18:20, on days 1, 5, 8 and 12 of the experiment. Results are expressed in µmoL electrons m^{-2} s^{-2}. Different colors represent the different treatments: yellow is amber, violet is amber +UV, blue is amber + blue and green is amber+ green.

Table S1. Multifactorial ANOVA effects for photosynthetic parameters extracted from Rapid Light Curves, including F_v/F_m, αETR, ETR_{max} and saturation irradiance (E_k), on day 7 and day 14, independently. Significant differences are shown in red ($p < 0.05$).

Day	Parameter	Factors	Degr. F	SS	MS	F	P
**7**	F_v/F_m	Radiation	3	0.006	0.002	0.80	0.511
		Nutrient	1	0.000	0.000	0.00	0.962
		Radiation * Nutrient	3	0.033	0.011	4.41	0.019
	αETR	Radiation	3	0.000	0.000	0.07	0.973
		Nutrient	1	0.000	0.000	0.39	0.541
		Radiation * Nutrient	3	0.002	0.001	1.63	0.222
	ETR_{max}	Radiation	3	1.024	0.341	0.30	0.828
		Nutrient	1	1.051	1.051	0.91	0.354
		Radiation * Nutrient	3	3.974	1.325	1.15	0.360
	E_k	Radiation	3	2260	753.4	0.12	0.945
		Nutrient	1	4387	4387	0.71	0.410
		Radiation * Nutrient	3	17903	5968	0.97	0.430
**14**	F_v/F_m	Radiation	3	0.009	0.003	0.88	0.470
		Nutrient	1	0.000	0.000	0.02	0.881
		Radiation * Nutrient	3	0.011	0.004	1.10	0.378
	αETR	Radiation	3	0.000	0.000	0.75	0.540
		Nutrient	1	0.001	0.001	4.41	0.052
		Radiation * Nutrient	3	0.000	0.000	0.81	0.508
	ETR_{max}	Radiation	3	5.780	1.927	1.59	0.232
		Nutrient	1	2.475	2.475	2.04	0.173
		Radiation * Nutrient	3	17.41	5.805	4.78	0.015
	E_k	Radiation	3	376.4	125.5	0.35	0.788
		Nutrient	1	2368	2367	6.65	0.020
		Radiation * Nutrient	3	1857	619.1	1.74	0.200

Table S2. Multifactorial ANOVA effect for *in situ* ETR on days 1, 5, 8 and 14, with measurements at 09:45, 15:00 and 18:20. Significant differences are shown in red ($p < 0.05$).

Day	Hour	Factors	Degr. F	SS	MS	F	P
**1**	09:45	Radiation	3	0.017	0.006	0.43	0.737
		Nutrient	1	0.042	0.042	3.10	0.098
		Radiation * Nutrient	3	0.043	0.014	1.06	0.394
	15:00	Radiation	3	0.069	0.023	0.23	0.875
		Nutrient	1	0.407	0.407	4.07	0.061
		Radiation * Nutrient	3	0.252	0.084	0.84	0.493
	18:20	Radiation	3	0.053	0.018	0.41	0.751
		Nutrient	1	0.295	0.295	6.75	0.019
		Radiation * Nutrient	3	0.084	0.028	0.64	0.598
**5**	09:45	Radiation	3	0.025	0.008	0.66	0.588
		Nutrient	1	0.066	0.066	5.32	0.035
		Radiation * Nutrient	3	0.026	0.009	0.69	0.574
	15:00	Radiation	3	0.2039	0.068	0.44	0.730
		Nutrient	1	0.000	0.000	0.00	0.986
		Radiation * Nutrient	3	0.845	0.282	1.81	0.187
	18:20	Radiation	3	0.194	0.065	1.73	0.201
		Nutrient	1	0.314	0.314	8.39	0.011
		Radiation * Nutrient	3	0.186	0.062	1.66	0.216
**8**	09:45	Radiation	3	0.076	0.025	1.32	0.303
		Nutrient	1	0.011	0.011	0.58	0.458
		Radiation * Nutrient	3	0.049	0.016	0.86	0.481
	15:00	Radiation	3	0.690	0.230	1.87	0.176
		Nutrient	1	0.384	0.384	3.12	0.097
		Radiation * Nutrient	3	0.239	0.080	0.65	0.596
	18:20	Radiation	3	0.276	0.092	1.59	0.232
		Nutrient	1	0.236	0.236	4.07	0.061
		Radiation * Nutrient	3	0.070	0.023	0.40	0.752
**12**	09:45	Radiation	3	0.001	0.000	0.03	0.992
		Nutrient	1	0.068	0.068	6.21	0.024
		Radiation * Nutrient	3	0.017	0.006	0.53	0.670
	15:00	Radiation	3	0.834	0.278	3.13	0.055
		Nutrient	1	0.003	0.003	0.03	0.861
		Radiation * Nutrient	3	0.182	0.061	0.68	0.576
	18:20	Radiation	3	0.094	0.031	1.11	0.374
		Nutrient	1	0.029	0.029	1.03	0.325
		Radiation * Nutrient	3	0.079	0.026	0.93	0.449

Table S3. Multifactorial ANOVA effect for NO_3 absorption, PO_4 absorption, Chlorophyll *a* (Chl *a*), Phycorythrin (PE), Phycocyanin (PC) and Mycosporine-like amino acids (MAAs) in *Plocamium cartilagineum* thalli on day 7 of the experiment. The significant differences ($p < 0.05$) are shown in red.

Parameter	Factors	Degr. of Freedom	SS	MS	F	P
**NO_3^- Absorption**	Radiation	3	1939.5	646.5	2.83	0.07
	Nutrient	1	14357.7	14357.7	62.79	0.001
	Radiation * Nutrient	3	2206.5	735.5	3.22	0.051
**PO_4^{3-} Absorption**	Radiation	3	89.2	29.72	0.46	0.71
	Nutrient	1	32.7	32.67	0.51	0.486
	Radiation * Nutrient	3	361.9	120.66	1.87	0.175
**Chl *a***	Radiation	3	47314	15771	5.09	0.011
	Nutrient	1	2185	2185	0.71	0.413
	Radiation * Nutrient	3	706	235	0.08	0.972
**Phenolic compounds**	Radiation	3	9.735	3.245	15.43	0.001
	Nutrient	1	0.958	0.958	4.56	0.048
	Radiation * Nutrient	3	2.451	0.817	3.89	0.029
**PE**	Radiation	3	13204858	4401619	5.94	0.006
	Nutrient	1	1154720	1154720	1.56	0.230
	Radiation * Nutrient	3	12962201	4320734	5.83	0.007
**PC**	Radiation	3	1712348	570783	4.49	0.019
	Nutrient	1	705942	705942	5.56	0.031
	Radiation * Nutrient	3	1299810	433270	3.41	0.043
**MAAs**	Radiation	3	1.506	0.502	2.55	0.092
	Nutrient	1	1.750	1.750	8.88	0.009
	Radiation * Nutrient	3	2.421	0.807	4.09	0.025

Table S4. Multifactorial ANOVA effect for NO_3^- absorption, PO_4^{3-} absorption, Chlorophyll *a* (Chl *a*), Phycorythrin (PE), Phycocyanin (PC) and Mycosporine-like amino acids (MAAs) in *Plocamium cartilagineum* thalli on day 14 of the experiment. The significant differences ($p < 0.05$) are shown in red.

Parameter	Factors	Degr. of Freedom	SS	MS	F	P
**NO_3^- Absorption**	Radiation	3	1121.8	373.9	13.78	0.001
	Nutrient	1	10895.1	10895.1	401.71	0.000
	Radiation * Nutrient	3	976.4	325.5	12.00	0.001
**PO_4^{3-} Absorption**	Radiation	3	294.16	98.05	2.44	0.102
	Nutrient	1	490.01	490.01	12.19	0.003
	Radiation * Nutrient	3	337.33	112.44	2.80	0.074
**Chl *a***	Radiation	3	114343	38114	1.89	0.171
	Nutrient	1	181264	181264	9.01	0.008
	Radiation * Nutrient	3	20913	6971	0.35	0.792
**Phenolic compounds**	Radiation	3	3.842	1.28	3.72	0.033
	Nutrient	1	1.209	1.21	3.51	0.079
	Radiation * Nutrient	3	0.740	0.25	0.72	0.556
**PE**	Radiation	3	3600722	1200241	2.55	0.092
	Nutrient	1	9385258	9385258	19.92	0.001
	Radiation * Nutrient	3	2774444	924815	1.96	0.160
**PC**	Radiation	3	496320	165440	3.90	0.029
	Nutrient	1	242496	242496	5.72	0.029
	Radiation * Nutrient	3	174263	58088	1.37	0.287
**MAAs**	Radiation	3	1.328	0.44	7.24	0.003
	Nutrient	1	0.301	0.30	4.89	0.042
	Radiation * Nutrient	3	1.373	0.46	7.48	0.002

Table S5. Multifactorial ANOVA effect for shinorine levels in *Plocamium cartilagineum* thalli on days 1, 7 and 14. The significant differences ($p < 0.05$) are shown in red.

Parameter	Factors	Degr. of Freedom	SS	MS	F	P
**Shinorine**	Radiation	3	0.052	0.0174	9.69	0.0001
	Nutrient	1	0.082	0.082	45.41	0.0001
	Time	1	0.062	0.062	34.36	0.0001
	Radiation * Nutrient	3	0.032	0.011	5.87	0.003

Radiation * Time	3	0.129	0.043	23.87	0.0001
Nutrient * Time	1	0.000	0.000	0.02	0.899
Radiation * Nutrient * Time	3	0.002	0.001	0.42	0.740
